# Directed evolution of P-glycoprotein cysteines reveals site-specific, non-conservative substitutions that preserve multidrug resistance

**DOI:** 10.1042/BSR20140062

**Published:** 2014-06-25

**Authors:** Douglas J. Swartz, Leo Mok, Sri K. Botta, Anukriti Singh, Guillermo A. Altenberg, Ina L. Urbatsch

**Affiliations:** *Department of Cell Biology and Biochemistry, Texas Tech University Health Sciences Center, Lubbock, Texas, U.S.A.; †Center for Membrane Protein Research, Texas Tech University Health Sciences Center, Lubbock, Texas, U.S.A.; ‡Department of Cell Physiology and Molecular Biophysics, Texas Tech University Health Sciences Center, Lubbock, Texas, U.S.A.

**Keywords:** ABC transporter, multidrug transporter, non-conservative cysteine substitutions, protein evolution site-saturation mutagenesis, yeast drug resistance, ABC, ATP-binding-cassette, CFTR, cystic fibrosis transmembrane conductance regulator, CL, Cys-less, CP-MTS, 7-diethylamino-3-(4′-maleimidylphenyl)-4-methylcoumarin, DDM, n-dodecyl-β-D-maltopyranoside, ICL, intracellular loop, NBD, nucleotide-binding domain, Pgp, P-glycoprotein, SEC, size exclusion chromatography, TMD, transmembrane domain, WT, wild-type

## Abstract

Pgp (P-glycoprotein) is a prototype ABC (ATP-binding-cassette) transporter involved in multidrug resistance of cancer. We used directed evolution to replace six cytoplasmic Cys (cysteine) residues in Pgp with all 20 standard amino acids and selected for active mutants. From a pool of 75000 transformants for each block of three Cys, we identified multiple mutants that preserved drug resistance and yeast mating activity. The most frequent substitutions were glycine and serine for Cys^427^ (24 and 20%, respectively) and Cys^1070^ (37 and 25%) of the Walker A motifs in the NBDs (nucleotide-binding domains), Cys^1223^ in NBD2 (25 and 8%) and Cys^638^ in the linker region (24 and 16%), whereas close-by Cys^669^ tolerated glycine (16%) and alanine (14%), but not serine (absent). Cys^1121^ in NBD2 showed a clear preference for positively charged arginine (38%) suggesting a salt bridge with Glu^269^ in the ICL2 (intracellular loop 2) may stabilize domain interactions. In contrast, three Cys residues in transmembrane α-helices could be successfully replaced by alanine. The resulting CL (Cys-less) Pgp was fully active in yeast cells, and purified proteins displayed drug-stimulated ATPase activities indistinguishable from WT (wild-type) Pgp. Overall, directed evolution identified site-specific, non-conservative Cys substitutions that allowed building of a robust CL Pgp, an invaluable new tool for future functional and structural studies, and that may guide the construction of other CL proteins where alanine and serine have proven unsuccessful.

## Abstract 2

Directed evolution of Cys (cysteines) in the multidrug transporter Pgp identified novel non-conservative substitutions, revealed important domain interactions and produced a CL protein that is more robust and active than previous CL Pgp built solely on conservative alanine substitutions.

## INTRODUCTION

Pgp (P-glycoprotein), also known as ABCB1 or MDR1, is a member of the ABC (ATP-binding-cassette) transporter superfamily that functions as a multidrug efflux pump [1,2]. It can contribute to multidrug resistance in cancers and other human diseases by preventing intracellular accumulation of cytotoxic agents, such as anticancer drugs [3,4]. Polyspecific binding of transported drugs and Pgp inhibitors occurs in a central cavity formed by two TMDs (transmembrane domains), but the precise locations and nature of these binding sites remain poorly defined [5,6]. Pgp and related ABC transporters have been crystallized in inward-facing and outward-facing conformations, which suggest an alternating access transport mechanism that is powered by association/dissociation of the two intracellular NBDs (nucleotide-binding domains) [6–9]. However, these conformations have not been well defined in solution. Identifying conformational changes that occur during the Pgp drug transport cycle, and understanding the molecular details of the drug/inhibitor binding sites will provide information that is necessary for structure-based design of novel Pgp inhibitors that could then be evaluated for clinical efficacy [10].

A variety of biophysical techniques have been applied to studying conformational changes and protein ligand interactions in membrane proteins including EPR and NMR spectroscopy, and RET (resonance energy transfer) using optical probes [11–14]. Such studies in Pgp would require a CL (Cys-less) protein for the introduction of site-specific Cys residues to avoid labelling of native Cys that are susceptible to covalent modification [15,16]. Previously, two CL human Pgp proteins were generated by replacing each native Cys with Ala or Ser [16,17]. These two CL proteins were found to be functional in mammalian cells, but did exhibit differences in drug-binding and drug-stimulated ATPase activity when compared with each other or WT (wild-type) human Pgp. CL human Pgps have been extensively used for covalent crosslinking and substrate-binding studies using native cell membranes and purified protein [18,19]. However, mammalian expression is not well suited for large-scale purification, limiting its application to biochemical and biophysical studies that require significant amounts of highly purified protein. In addition, human Pgp protein yield and stability is lower than that of the mouse Pgp orthologue expressed in heterologous yeast expression systems [20,21]. To avoid this limitation, a CL mouse Pgp where all Cys were replaced with Ala was constructed and expressed in yeast [22]. While this catalytically active CL mouse Pgp can be purified from *Pichia pastoris* yeast in reasonable quantities, its function is diminished. In addition, drug resistance that it confers to *Saccharomyces cerevisiae*, *in vivo*, is greatly impaired. Here, we undertook steps to generate a new fully functional, highly expressed mouse CL Pgp that will serve as an improved tool for biophysical studies of the Pgp transport mechanism.

**Figure 1:**
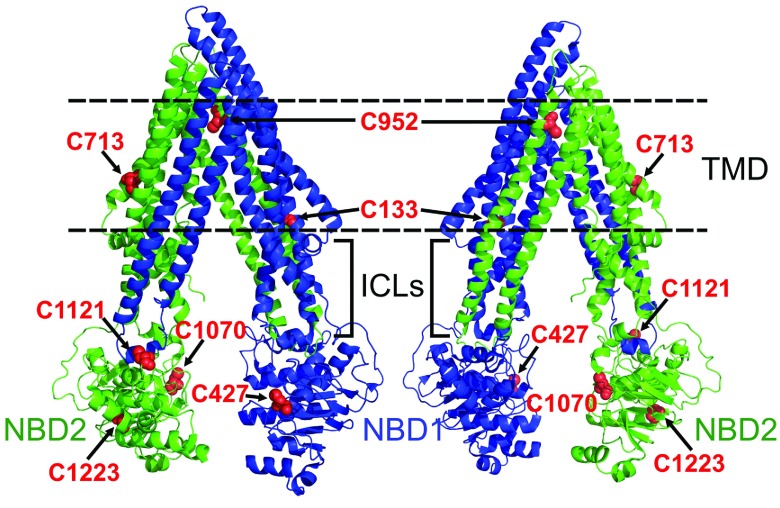
Location of the nine native Cys residues in Pgp Cys residues (red) are shown in two views of the Pgp crystal structure (3G61). Cys^427^ is located in the N-terminal NBD (NBD1, blue), while Cys^1070^, Cys^1121^ and Cys^1223^ are in the C-terminal NBD (NBD2, green). Cys^133^, Cys^713^ and Cys^952^ are located in the membrane bound portion of the TMDS. Cys^638^ and Cys^669^ are in the flexible linker between NBD1 and TMD2 that was not resolved in the Pgp crystal structure. Dotted lines delineate the approximate location of the membrane bilayer. ICL, intracellular loop.

CL proteins have been created for a variety of membrane transporters by the common strategy of replacing native Cys residues with Ala or Ser, but replacement of native Cys can often result in partial or complete loss of protein expression and/or function, as with Pgp [23–28]. A study by Ullman and co-workers demonstrated that site-saturation mutagenesis could be used to identify functional Cys substitutes, for individual Cys residues that could not be successfully replaced by Ala or Ser [29]. Their work suggests that Ala and Ser are not always the ideal Cys replacement. Mouse Pgp contains nine native Cys residues (2 more than human Pgp), with three located in the TMDs, three in the N-terminal NBD/linker region, and three in the C-terminal NBD (Figure 1). Our study uses a directed evolutionary approach to replace the six cytoplasmic Cys residues with all 20 standard amino acids and selecting for active mutants. Mutants were constructed using the codon optimized Pgp gene that we previously found to increase yield and quality of Pgp expressed in yeast [30]. We identified multiple Cys mutants that were as active as WT Pgp and maintained Pgp substrate polyspecificity, but contained non-conservative substitutions at six sites. The resulting CL-Pgp maintained the expression and full activity in yeast functional assays, and the purified protein displayed drug-stimulated ATPase activity indistinguishable from that of WT Pgp. Overall, directed evolution identified novel non-conservative cysteine substitutions, revealed important domain interactions and produced a CL protein that, because it is much more robust and active than previous versions, will be an invaluable new tool for studying the Pgp mechanism.

## EXPERIMENTAL

### Materials

FK506, cyclosporine A, doxorubicin and valinomycin were purchased from A.G. Scientific. Fluconazole was from LKT Laboratories. Verapamil, nystatin and ATP were from Sigma Aldrich. *Escherichia coli* lipids (Polar Extract) were purchased from Avanti Polar Lipids, and DDM (n-dodecyl-β-D-maltopyranoside) was from Inalco. CP-MTS [7-diethylamino-3-(4′-maleimidylphenyl)-4-methylcoumarin] was from Biotium.

### Mutant construction

**Figure 2:**
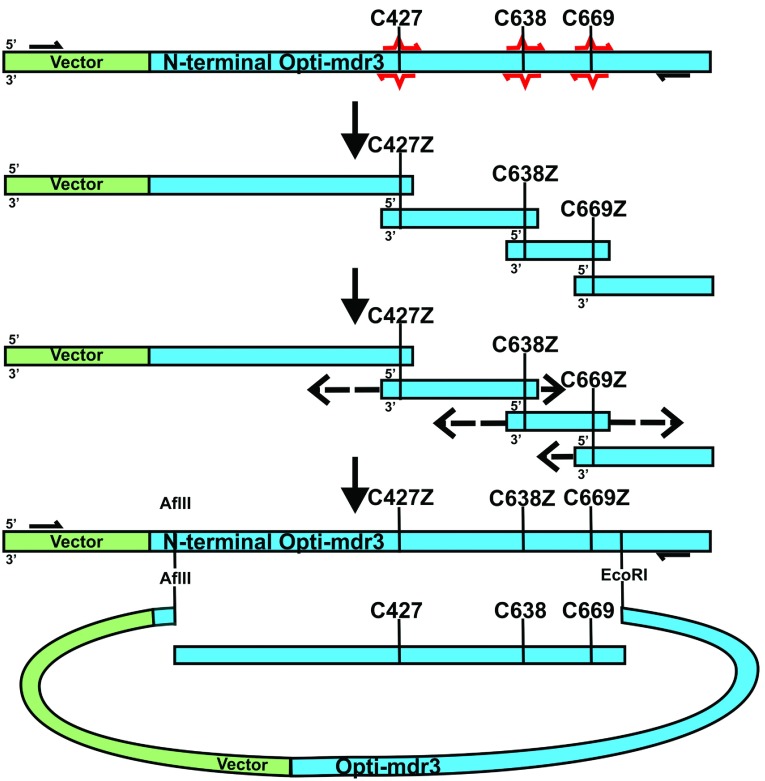
Strategy for construction of the C427Z, C638Z and C669Z mutant block by site-saturation mutagenesis An overlap extension PCR approach is used to amplify initial fragments using mutagenic (red arrows) and flanking primers (black arrows) with pVT-opti-mdr3 as template. Gel-purified fragments are annealed and PCR assembled with the flanking primers to generate a fragment containing appropriate unique restriction sites (in this case 5′- AflII and 3′- EcoRI). Gel purified PCR fragments and vector (with the AflII-EcoRI fragment excised) are then cotransformed into yeast for assembly via homologous recombination.

The C427Z/C638Z/C669Z (NBD1/linker) and C1070Z/C1121Z/C1223Z (NBD2) mutant blocks were generated by site-saturation mutagenesis using oligonucleotides that contained a fully degenerate codon at each Cys position (NNN, where N represents an equal mixture of A, T, G and C nucleotides), as outlined in Figure 2 (Supplementary Table S1 at http://www.bioscirep.org/bsr/034/bsr034e116add.htm). A codon-optimized mouse Pgp (*mdr1a*, *mdr3*, GenBank JF834158) in the pVT expression vector (pVT-Opti-mdr3) served as the PCR template to enhance yeast expression and production of high-quality protein [30]. This optimized Pgp has the same amino acid sequence and is functionally identical to non-optimized Pgp, and is referred to as WT Pgp. Degenerate codons were introduced by overlap extension PCR using Phusion Hotstart II polymerase (Thermofisher) [31]. First, mutagenic and non-mutagenic flanking primers were used by PCR to amplify four individual fragments that overlapped at the three Cys sites in each block. The four fragments were resolved on a 2% (w/v) agarose gel and purified by electroeluting DNA from excised agarose fragments in 12–14 kDa MWCO (molecular-mass cut-off) Spectrapor dialysis membrane (Spectrum) in TAE (Tris/acetate/EDTA) buffer. Each purified fragment (100 ng) was combined and extended in a second PCR reaction without additional PCR primers; then, flanking primers were added to amplify the full-length block. The flanking primers extended the NBD1/linker block by approximately 100 bp beyond an upstream AflII restriction site and a downstream EcoRI restriction site, to allow for homologous recombination with digested, gel-purified pVT-Opti-mdr3 plasmid in yeast [32]. Similarly, the NBD2 cysteine block containing C1070Z/C1121Z/C1223Z was generated with flanking primers that extended ~100 bp beyond the upstream SpeI site and the downstream AgeI site for homologous recombination. The NBD1/linker and NBD2 mutant blocks were co-transformed with their respective digested pVT-Opti-mdr3 plasmids into competent *S. cerevisiae* JPY201 (MATa ura3 Δste6::HIS3) cells by electroporation [33,34]. Usually, ~1 μg of cut plasmid was co-transformed with a 2-fold excess of PCR fragment per electroporation sample, yielding >3000 colonies; a total of 15 electroporations were performed for each block to obtain approximately 75000 transformants.

### Selection and screening methods

Transformants were plated on uracil-deficient medium (uracil is the selection marker for the pVT vector [35] containing 150 μM fluconazole to start the selection process. Parallel samples were plated on uracil-deficient medium (without fluconazole) to estimate transformation efficiencies. Colonies from fluconazole containing plates were combined into mass population stocks, and a 1/20 volume of the stocks was inoculated into 15 ml liquid cultures containing 2.5 μM nystatin. The cultures were then grown to density in a shaking incubator at 30°C for 3–4 days. The resulting cultures were diluted 30-fold into 15 ml of fresh medium containing 150 μM fluconazole and again grown to density, followed by successive dilutions and growths in nystatin and a combination of doxorubicin (15 μM) and valinomycin (50 μM). Vector controls (transformed with empty pVT vector), and WT strains were grown in parallel to assess selection efficiency. Finally, the cells were allowed to recover for 3 h by growth in the drug-free medium and mated with an α-type yeast strain DC17 to identify those that restore mating in the otherwise sterile ste6Δ yeast strain JPY201 [34]. To identify the Cys mutations in yeast that successfully mated, DNA from single colonies was amplified by PCR in 96-well plates using the appropriate flanking primers for each block (Supplementary Table S1) and the PCR fragments sequenced (Beckman Coulter Genomics) [31]. Colonies picked for sequencing were maintained on uracil-deficient agar plates (one-well plates to track the PCR well numbers) and stored at 4°C. Plasmids of unique mutants were recovered from 10 ml yeast cultures by disrupting the cells with glass beads, purifying the plasmid DNA using a Wizard Plus miniprep Kit (Promega) and propagating extracted plasmids in XL10 Gold *E. coli* cells (Stratagene), followed by full-length sequencing.

### Combining mutant blocks

Homologous recombination was also used to combine blocks of NBD1/linker and NBD2 Cys mutants. Plasmids containing NBD1/linker Cys mutants were digested with SpeI and AgeI and gel-purified. NBD2 Cys mutant blocks were PCR amplified with the flanking primers using single colonies as templates. The gel-purified fragments were pooled and co-transformed into yeast with the digested NBD1/linker plasmids. To confirm the presence of NBD2 mutants, single yeast colonies were amplified by PCR and digested with PstI; a unique PstI site is removed in all C1070Z mutants (Z≠C). A total of 42 single colonies were screened in yeast for biological activity. Then, plasmids were extracted again, and unique mutant combinations were confirmed by full-length sequencing before being retransformed into naïve *S. cerevisiae* for detailed analysis. C133A, C713A and C952A were generated by site-directed mutagenesis (QuikChange II XL, Stratagene) using the oligonucleotide primers given in Supplementary Table S1 to generate the fully CL pVT-CL-Opti-mdr3.

### Analysis of Cys mutant expression and function

To assess mutant function, yeast fungicide resistance assays were performed as previously described [30,36]. Briefly, 10 ml yeast cultures were grown overnight in uracil-deficient medium containing 7.5% (v/v) glycerol, diluted to OD600=0.05 in YPD medium [1% (w/v) yeast extract/2% (w/v) peptone/2% (w/v) glucose], and seeded into 96-well plates containing YPD alone or YPD plus 50 μM FK506, 100 μM valinomycin or 40 μM doxorubicin. Samples were grown in triplicate at 30°C for up to 30 h, and yeast cell growth was monitored by measuring the OD600 at 2 h increments in a microplate reader (Benchmark Plus, BioRad). The remainder of the 10 ml cultures was used to assess Pgp expression by the Western blot analysis of microsomal membrane preparations as described [20].

### WT and Cys mutant purification and analysis

For large-scale protein purification, JPY201 cells expressing WT and mutant Pgps were grown in 14 litre fermentor cultures (BioFlow IV) of uracil-deficient medium containing 7.5% glycerol, and harvested during log-phase growth (OD600 ~4.0). Microsomal membranes were processed as previously described except that the membranes were washed with 500 mM NaCl to remove peripheral proteins and increase final protein purity [20,30]. Pgp was extracted with 0.6% DDM in the presence of 250 mM NaCl and purified by nickel affinity chromatography and SEC (size exclusion chromatography) as described for *P. pastoris* expressed Pgp [20,30], except that the NaCl concentration of all buffers was increased from 50 to 250 mM to increase protein stability [20,30]. Pgp concentrations were initially determined from the absorbance at 280 nm (A280) using a calculated molar extinction coefficient of 109,750 M-1 cm-1 (1 A280 unit=1.29 mg/ml). WT and Cys mutants were resolved side-by-side on SDS/PAGE gels, stained with Coomassie Brilliant Blue and Pgp was quantified using ImageJ (http://rsbweb.nih.gov) to compare mutant Pgp levels; Pgp purified from *P. pastoris* served as standard. For ATPase assays, purified Pgp was activated with 10 mM DTT (dithiothreitol) and 1% *E. coli* polar lipids and ATPase activity was measured at 37°C in a coupled assay utilizing an ATP-regenerating system, as previously described [30,37]. Drug-stimulated ATPase activity was analysed by the equation V=Vbas+ (Vmax×S^b^)/(S^b^+Ks^b^), where S is the concentration of stimulatory drug, V is the rate of ATP hydrolysis, Vbas is the ATPase activity in the absence of drug, Vmax is the maximum drug stimulated ATPase activity, Ks is the concentration of drug required for half-maximal stimulation, and b is the Hill coefficient. Cyclosporine A inhibition was analysed using the equation V=Vmax-(Imax×I^b^)/(Ki^b^+I^b^), where I is the inhibitor concentration, Imax is the maximal inhibited ATPase activity, and Ki is the inhibitor concentration required for half-maximal inhibition.

## RESULTS

### Substitution of intracellular Cys residues

Each of the nine native Cys residues in mouse Pgp is highly conserved among Pgp orthologues except Cys^638^ and Cys^669^, both located in the flexible linker that connects the two homologous halves of the protein (Supplementary Table S2 at http://www.bioscirep.org/bsr/034/bsr034e116add.htm). Four of the six cytoplasmic Cys residues are located in the highly conserved NBDs that contain motifs necessary for ABC transporter function (Figure 1) [2,15]. In contrast, the three Cys in the TMDs are located in highly ordered transmembrane helices where the small, hydrophobic methyl Ala side chain would likely be a good substitution because it supports the α-helical structure [38]. Therefore our first objective in this study was to replace the six cytoplasmic Cys by site-saturation mutagenesis, allowing every possible amino acid substitution at every Cys position, and determine which amino acids were the most likely to retain activity. Ideally, all six native Cys would be replaced simultaneously to account for potential structural distortions within the domains as well as interdomain communication (Figure 1). However the large number of possible combinations (20^6^=64×10^6^) is impractical to screen. Instead, we simultaneously replaced the three Cys in NBD1 and the adjacent linker region (Cys^427^, Cys^638^, Cys^669^) in one construct, and the three Cys in NBD2 (Cys^1070^, Cys^1121^ and Cys^1223^) in a second construct, reducing the required number of mutants (20^3^ amino acids=8000 per block of three, or 64^3^ codons=262000). For site-saturation mutagenesis, an overlap extension PCR approach was used to replace the native Cys codons with fully degenerate (NNN) codons that encode all 20 amino acids (64 codons), including Cys (Figure 2). Mutant PCR libraries were directly transformed into *S. cerevisiae* by homologous recombination (see the Materials and Methods section for details) to select for active mutants by successive passages in different fungicides (nystatin, fluconazole, valinomycin and doxorubicin) followed by a mating assay. This selection scheme is based on the ability to convey resistance of the yeast to fungicides as well as export a-factor pheromone and restore mating in the otherwise sterile yeast by complementing for Ste6, a Pgp yeast homologue [34,39]. Mating, by itself, is a stringent test of mutant function as we previously demonstrated [40]. The customized selection scheme was designed to identify mutants that could transport multiple, structurally diverse substrates and preserve polyspecificity, an important quality of Pgp.

**Figure 3:**
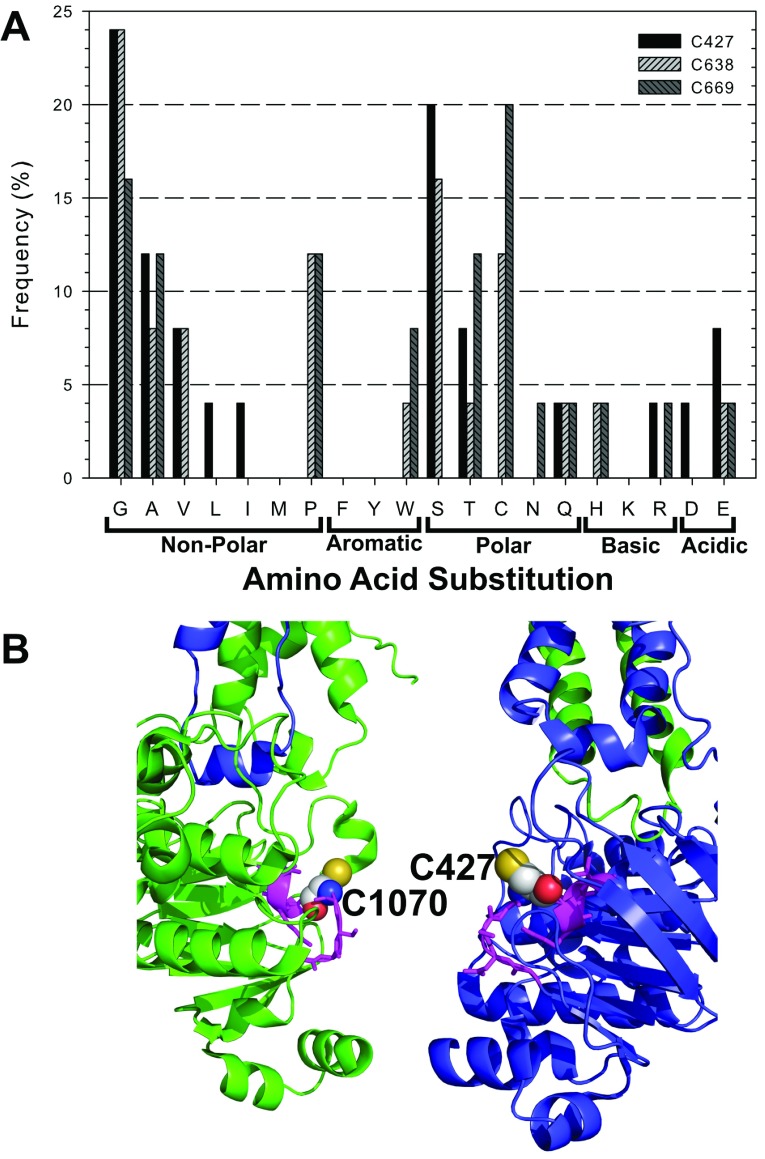
Frequency of NBD1/linker amino acid substitutions (**A**) The frequency of each amino acid found at the three Cys positions in 25 unique NBD1/linker Cys mutants identified from sequencing of yeast colonies after selection. (**B**) Location of Cys^427^ in NBD1 (blue) and Cys^1070^ in NBD2 (green) of Pgp. The other amino acids in the Walker A motifs are shown in magenta.

**Figure 4:**
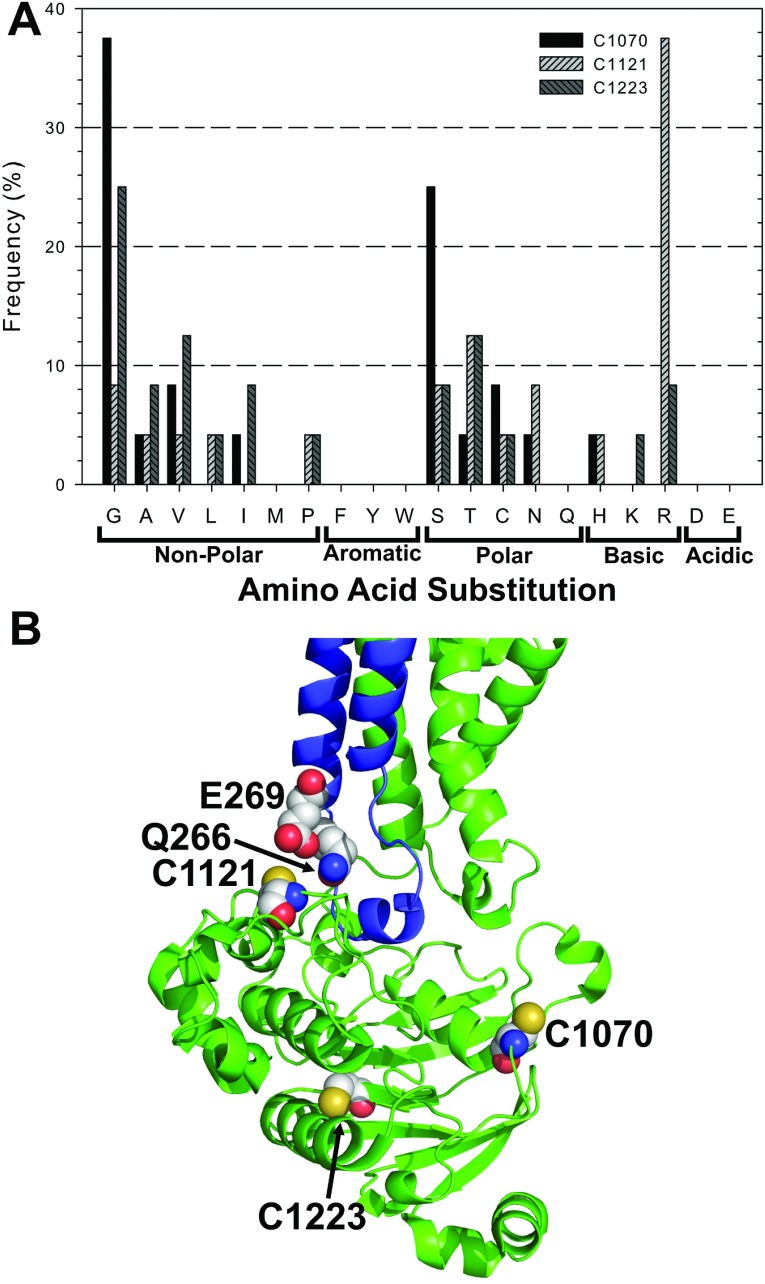
Frequency of NBD2 amino acid substitutions (**A**) The frequency of each amino acid substitution found at the three Cys sites in 24 unique NBD2 Cys mutants. (**B**) Location of the Cys residues in NBD2 (green). A Glu and Gln, from the N-terminal ICL (blue), face Cys^1121^ which was frequently replaced by the positively charged amino acid Arg.

In total, approximately 75000 transformants carrying the NBD1/linker Cys mutants entered the selection process and 25 unique triple Cys mutant combinations were recovered after yeast mating. Likewise, 75000 transformants carrying the NBD2 Cys mutants passed through the selection process and 24 unique mutants were recovered. Unique mutants were defined as a distinct combination of amino acids as well as distinct codons across the Cys sites in a mutant block, or mutants with the same combination of codons that were derived from different transformation/selection samples. Analysis of the codon usage revealed an overall bias (>70%) towards low-frequency codons (defined as those occurring at <10% frequency in highly expressed yeast genes [30,41]) for all of the six Cys positions, possibly because Cys is an amino acid that occurs at low frequency (nine Cys per 1276 amino acids in Pgp). Frequencies of amino acid substitutions for each Cys site are given in Figures 3 and 4, and are summarized below according to the locations of the Cys in the protein.

### Cysteine substitutions found by directed evolution

For Cys^427^, located in the Walker A motif of NBD1, Gly was the most prominent substitution occurring at a frequency of 24%, followed by Ser at 20% (Figure 3A). Other small hydrophobic amino acids, Ala (12%) and Val (8%) and the polar Thr (8%) were also present. A similar pattern was observed for the Walker A motif Cys^1070^ of NBD2, with Gly (37%) being the most prominent substitution, followed by Ser (25%). A difference was that Ala and Thr occurred at very low frequencies of just 4% (Figure 4A). The Walker A motif is also called glycine-rich loop (or P-loop) because it wraps around the phosphates of bound ATP and positions the γ-phosphate for hydrolysis (Figure 3B). Thus small hydrophobic Gly or polar Ser substitutions may support better the flexibility of this loop.

For the two Cys residues in the linker region, Cys^638^ and Cys^669^, many revertants were observed (12 and 20%, respectively). The most prominent substitutions for Cys^638^ were Gly (24%), Ser (16%) and Pro (12%) while Ala (8%) and Val (8%) substituted at lower frequencies. A different profile was observed for Cys^669^ with Gly, Ala, Pro and Thr all occurring at 12 to 16%, whereas Ser was absent. The linker is considered to be a highly flexible region that connects NBD1 to the TMD of the C-terminal Pgp half, which may explain a preference for small residues. No structural information is available on those Cys since this region was not resolved in the crystal structure (Figure 1).

For Cys^1121^, we found a clear preference for Arg (38%) while Thr (13%), Ser (8%), Gly (8%) and Asn (8%) substituted but at low frequencies. In the crystal structure of Pgp, Cys^1121^ is located at the surface of NBD2 in close proximity to the ICL2 (intracellular loop 2) that extends from TMD1 and interfaces with NBD2 (blue, Figures 1 and 4B). In this arrangement, Cys^1121^ in NBD2 would face Glu^269^ and Gln^266^ in ICL2. This may provide an explanation for the preference of a positively charged Cys substitution here. For Cys^1223^, Gly was the most common replacement (25%), while Ala, Val, Ile, Ser, Thr and positively charged Arg all substituted at frequencies of 8 to 12%. Cys^1223^ lies within a β-sheet in the core of NBD2 where space restrictions may favour a preference for small residue substitutions, with Arg posing an exception.

### Screening and combining NBD1/linker and NBD2 Cys mutations

**Figure 5:**
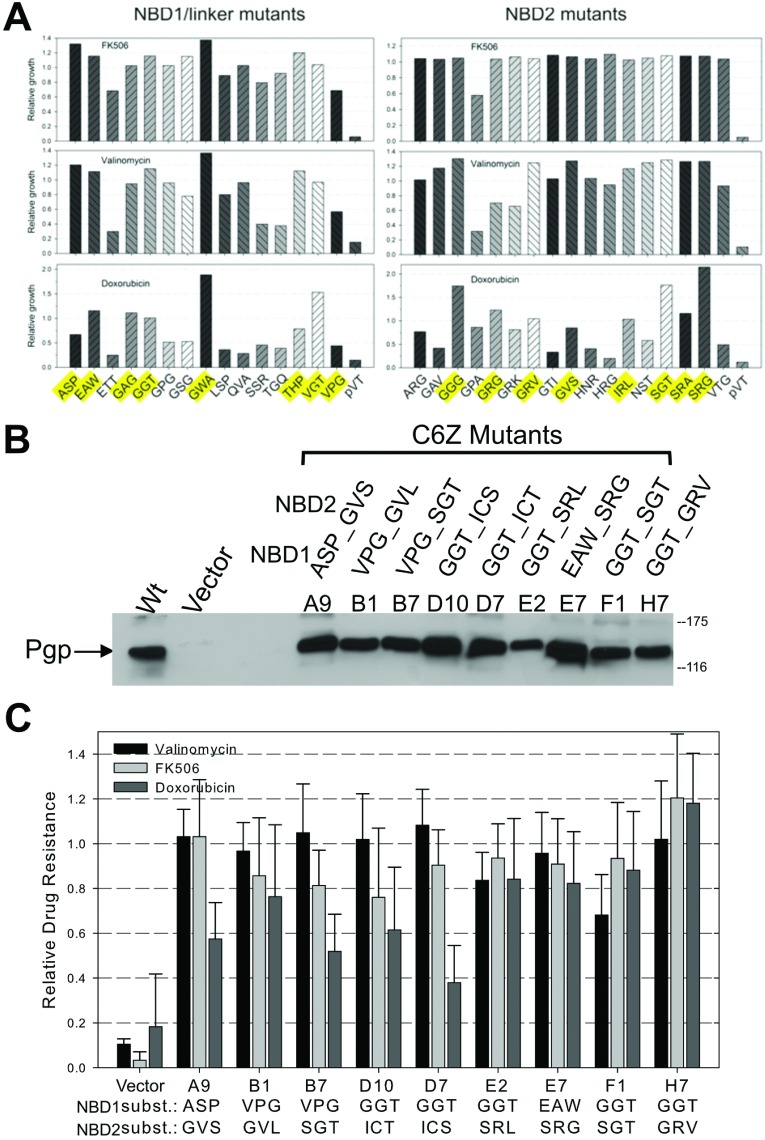
*In vivo* function of NBD1/linker and NBD2 Cys mutants (**A**) NBD1/linker and NBD2 Cys mutants were screened for resistance to 100 μM valinomycin, 50 μM FK506, and 40 μM doxorubicin. Relative resistance was calculated from the growth of each sample in drug compared with drug-free medium and then normalized to the relative growth of WT Pgp expressing yeast (WT=1). Amino acid substitutions are given in single letter code, e.g. ASP is C427A/C638S/C669P (left) and ARG is C1070A/C1121R/C1223G (right). A representative of two experiments is shown. Highlighted mutants were used to assemble the two mutant blocks. (**B**) Eight NBD1/linker and nine NBD2 unique mutants (highlighted in **A**) were combined as described in the Materials and Methods section, then plasmids extracted and retransformed into naive yeast cells for analysis of Pgp expression and function. 15 μg of crude microsomal membrane protein from each mutant expressing yeast strain and yeast expressing WT Pgp and empty pVT vector was analysed by Western blot using the Pgp-specific monoclonal antibody Cys^219^. Labels indicate the well number of the mutants from screening (A9 to H7), and the substitutions for the three consecutive Cys positions in NBD1 and NBD2 are given as single letter amino acid abbreviation. (**C**) The same nine mutants were tested for resistance against multiple drugs as in (**A**). Bars represent the mean of multiple replicates from three independent experiments±S.D..

After identifying mutants that successfully mated, a random set of 15 unique NBD1/linker and 17 unique NBD2 mutants were tested for their ability to convey resistance against three structurally diverse fungicides, FK506 (804 g/mol), valinomycin (1111 g/mol) and doxorubicin (544 g/mol) (Figure 5A). We did observe differences in drug resistance profiles between the three compounds even though all of the mutated Cys positions are located in the intracellular portions of the protein, distal to the drug-binding sites. In general, most mutants were highly resistant against FK506, which was not part of the drug selection regimen (≥80% growth compared with WT after 24 h, they did reach confluence after 26 h), as well as valinomycin, with a few exceptions (Figure 5A). The most pronounced differences were seen with doxorubicin as several mutants displayed enhanced growth compared with WT, while others grew slower, reaching only ~50% of the WT Pgp value after 24 h (they did grow to confluency after 30 h). Importantly, all mutants were active even against a drug that was not part of the selection (FK506), validating our selection scheme.

Eight NBD1/linker and eight NBD2 mutants that maintained the highest levels of resistance against all three fungicides (highlighted in Figure 5A) were chosen to be randomly assembled into mutants with all six intracellular Cys removed (C6Z). Forty two mutant yeast colonies were initially tested for fungicide resistance to screen for incompatibilities between the NBD1/linker and NBD2 mutations. Then, plasmid DNA for the 19 most active mutants was extracted and retransformed into naïve yeast for detailed analysis. Overall, expression levels of the combination mutants were similar to WT (Figure 5B). Most mutants retained high levels of drug resistance against valinomycin and FK506 (at least 80% growth compared with WT) (Figure 5C). The most pronounced differences were again seen with doxorubicin. Several mutants reached only 40–60% of the WT value after 24 h, whereas others, E2, E7 and particularly H7, were as active against the three drugs as WT Pgp. Several mutants were identified twice in our screen of randomly recombined mutants (A9, B1, B7, D7, E2 and E7), and F1 and H7 occurred three-times. H7 was named C6Z-Pgp and chosen as template for further mutagenesis. These results demonstrate that random recombination of NBD1/linker and NBD2 Cys mutations, identified by directed evolution successfully yielded several Pgp proteins that maintain high levels of activity with six native Cys residues removed.

**Figure 6:**
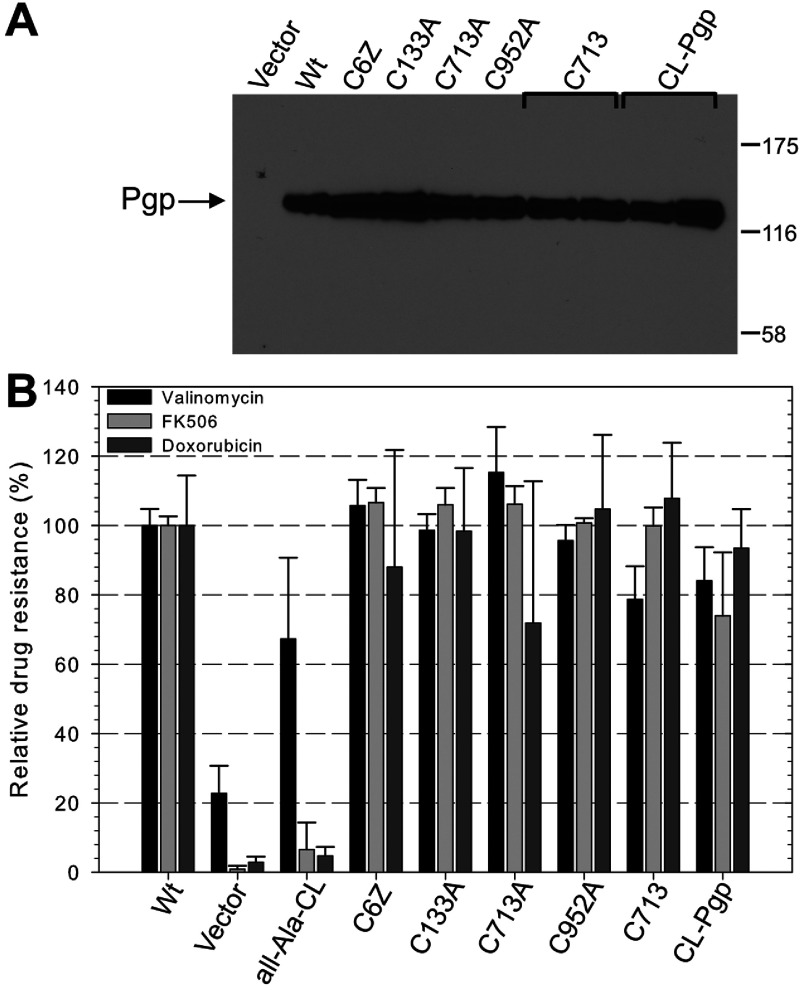
Expression and *in vivo* transport activity of CL and low Cys Pgp mutants (**A**) Western blot analysis of the diverse mutants was done as in Figure 5 with 15 μg of microsomal membrane protein loaded per lane (**B**). The CL and low-Cys mutant expressing strains were tested for fungicidal resistance as in Figure 5. C6Z- Pgp (H7 from Figure 5C) has all six cytoplasmic Cys removed and retains three native Cys in the TMDs. C133A, C713A and C952A each retain two native Cys in the TMDs, while C713 retains only one native Cys. CL-Pgp is fully CL with all nine Cys removed. All-Ala-CL, with all Cys mutated to Ala in the WT Pgp background, was included for comparison. Bars indicate the mean relative growth of samples normalized to the relative growth of WT Pgp expressing yeast from three independent experiments±S.D., nine independent experiments for CL-Pgp.

*Removal of TMD cysteines and construction of a CL Pgp–* Our second objective was to remove the remaining Cys residues in the TMDs (Cys^133^, Cys^732^, Cys^952^; Figure 1), individually and successively, from the C6Z-Pgp mutant. We chose Cys to Ala substitutions because Ala is known to stabilize α-helical structure [38]. Western blots revealed that all TMD Cys-to-Ala mutants maintained expression when compared with WT and C6Z (Figure 6A). All single and double TMD Cys mutants also maintained high levels of resistance to fungicides (Figure 6B), suggesting that Ala is a good substitution for Cys in transmembrane α-helices of Pgp. When all three TMD Cys mutations were added to the C6Z mutant to form a fully CL protein (CL-Pgp), the resulting protein exhibited at least 75% resistance against all three drugs relative to WT. For comparison, the mouse CL Pgp previously made by replacing all nine Cys with Ala in the non-codon-optimized Pgp background (‘all-Ala-CL’) was also expressed in *S. cerevisiae* yeast and assayed in parallel (Figure 6B) [22]. The all-Ala-CL Pgp only conferred partial resistance against valinomycin and none against FK506 and doxorubicin, demonstrating the value of the new CL-Pgp that was generated by directed evolution.

### Purified CL Pgp as background for thiol-selective labelling

**Figure 7:**
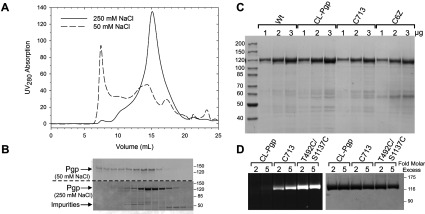
Purification and thiol-selective labelling of Cys mutants (**A**) Cl-Pgp was purified from *S. cerevisiae* fermentor cells by affinity chromatography on Ni-NTA resin as detailed in the Materials and Methods section, then 2–3 mg (500 μl) loaded on a Superose 6B column and resolved in buffers containing 50 or 250 mM NaCl. (**B**) SEC fractions were resolved on SDS-gels and stained with Coomassie Brilliant Blue. Molecular mass protein markers are given in kDa. Mutants Cys^713^ and C6Z-Pgp, or WT Pgp purified and chromatographed in 250 mM NaCl buffers gave very similar elution profiles as Cl-Pgp (not shown). (**C**) Increasing amounts of each purified protein were resolved on 10% SDS/PAGE gels and stained with Coomassie Brilliant Blue. (**D**) CL-Pgp, Cys^713^ and a control double Cys mutant T492C/S1137C were labelled for 1 h at room temperature with a 2-fold and 5-fold molar excess of the methanethiosulphonate reactive dye of coumarine (CP-MTS) as described, resolved on an SDS-gel and imaged at 385 nm (left), or stained with Coomassie Brilliant Blue (right). T492C/S1137C was generated by site-directed mutagenesis in the pVT-CL-opti-mdr3 background.

CL-Pgp, WT and selected Cys mutants were purified by affinity and SEC as described, except that NaCl (250 mM) was included in all buffers, as higher salt concentration have been shown to improve purification of human Pgp (see the Materials and Methods section for details) [30,42]. Proteins eluted in a major peak (at ~15.1 ml), consistent with an apparent size of ~200 kDa (monomeric protein), and a minor peak at ~13 ml that corresponded to a Pgp oligomer (Figure 7A). In contrast, when CL-Pgp (or WT, not shown) were chromatographed in 50 mM NaCl buffer the proteins eluted as several broad peaks in early fractions (9–14 ml) as well as in the void volume (Figures 7A and 7B), suggesting Pgp oligomerization and aggregation in low-salt buffers, contrary to *P. pastoris*-purified Pgp [30]. Purity of all Cys mutants was comparable with that of WT -Pgp, with minimal degradation products observed on Coomassie Brilliant Blue-stained SDS-gels (Figure 7C) or Western blots (not shown). The yield of the SEC-purified proteins was about 2.4 mg/100 g yeast cells from 14 litres fermentor cultures.

One major use of a the functional CL-Pgp is that it can be employed as framework to introduce single- and double-Cys mutants at selected positions for labelling with optical probes for spectroscopic studies. To demonstrate the usefulness of the CL Pgp as background for labelling studies, we compare it with a single (Cys^713^) and a double -Cys mutant (T492C/S1137C) that, as previously shown, has two very accessible cysteines in the NBDs [43]. Cys^713^ was generated as part of our site-directed mutagenesis aimed at substituting the last three TMD Cys (see above). For the labelling studies, Cys^713^ was reacted with the thiol-reactive coumarin dye, CP-MTS (Figure 7D). CL-Pgp and mutant T492C/S1137C were subjected to the same labelling protocol. Minimal fluorescence was detected in the CL-Pgp band on SDS-gels while Cys^713^ clearly showed fluorescence, and fluorescence increased in the T492C/S1137C as expected from the number of cysteines present (Figure 7D). The data indicate that the transmembrane Cys at position 713 is accessible to thiol reagents. In the crystal structure, the Cys^713^ sulfhydryl is oriented towards neighbouring α-helices (Figure 1) and it is therefore likely that it is not fully labelled. However, the low CL-Pgp background and the high sensitivity of fluorescence techniques allow for easy detection of the single-Cys. These results demonstrate the usefulness of the functional CL-Pgp as background for site-specific labelling of Cys residues introduced at selected positions in the protein.

### ATPase activity of CL-Pgp

**Figure 8:**
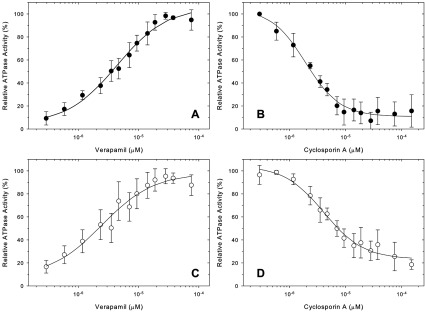
Stimulation and inhibition of ATPase activity of CL-Pgp and WT Pgp (**A**) WT and (**C**) CL-Pgp ATPase activity was assayed with purified proteins in the presence of increasing concentrations of verapamil. The solid lines represent non-linear regression analyses to the data points (see the Materials and Methods section) No cooperativity was observed with Hill coefficients close to 1.0 (1.04 and 1.00, respectively). Each data point represents the mean from at least three independent experiments±S.D.. (**B**) WT and (**D**) CL- Pgp purified proteins were assayed in the presence of 30 μM verapamil to maximally stimulate ATPase activity, but with increasing concentrations of the inhibitor cyclosporine A. The solid lines are non-linear regression fits, Hill coefficients were 1.40 and 1.22, respectively.

Purified proteins were assayed for drug stimulation or inhibition of ATPase activity, which is a benchmark test for comparing purified Pgp proteins [40,44]. Maximum verapamil-stimulated ATPase activity was similar for CL-Pgp, C6Z-Pgp and WT with 3.2±0.6, 3.5±0.2 and 3.5±0.7 μmol/min/mg ATP hydrolysed, respectively (Table 1). This value is somewhat higher than that of *P. pastoris*-purified Pgp (2.1±0.3 μmol/min/mg) [30], suggesting that increased salt is also beneficial for ATPase activity. The half-maximal stimulatory concentrations for verapamil were 4.8 μM, 2.0 and 3.8 μM for CL-Pgp, C6Z-Pgp and WT, respectively (Figures 8A and 8C; Table 1), not significantly different in the two tailed *t* test (*P* > 0.5). Inhibition of the verapamil-stimulated ATPase activity by the immunosuppressant cyclosporine A was also comparable for the two proteins, with half-maximal inhibition at 4.8 μM, 3.4 and 2.0 μM for CL-Pgp, C6Z-Pgp and WT, respectively (*P* > 0.05; Figures 8B and 8D; Table 1). Taken together, the enzymatic data indicate unaltered affinities for substrates and inhibitors between WT, C6Z-Pgp and CL-Pgp.

**Table 1:** ATPase activity of CL-Pgp and low-Cys mutants

Protein	ATPase activity* (μmole Pi/mg/min)	Ks Verapamil (μM) †	Ki Cyclosporine A (μM)‡
Wt	3.5±0.66	4.8±1.8	2.0±0.8
C6Z	3.5±0.19	2.0±1.1	3.4±0.9
CL-Pgp	3.2±0.56	3.8±3.3	4.8±2.3

*Maximum verapamil-stimulated ATPase activity in the presence of 30 μM verapamil (mean±S.E.M.).

†Concentration required for half-maximal ATPase activity derived from plots of V versus [drug] in Figures 8A and 8C (mean±S.E.M.); similar plots were obtained for C6Z-Pgp (not shown).

‡Cyclosporine A concentration required for half-maximal inhibition of verapamil-stimulated ATPase in Figures 8B and 8D (mean±S.E.M.); similar plots were obtained for C6Z-Pgp (not shown).

## DISCUSSION

This study employed a directed evolutionary approach to determine the preferred amino acid substitutions for Cys in the cytoplasmic domains of Pgp (Figures 3 and 4) to generate several highly-active low-Cys mutants with six Cys residues removed (Figure 5). We initially focused on the cytoplasmic Cys residues because they are located in the highly conserved, complexly folded NBDs where directed evolution could identify beneficial non-conservative substitutions and domain–domain interactions (Figure 1) [2,15]. Then, the three Cys in the transmembrane α helices were replaced with Ala, as the small non-polar side chain is known to support α-helical structure [38]. Substitution of these Cys residues by Ala resulted in a CL protein that was well expressed, displayed high levels of resistance against multiple fungicides, and could be purified in yields suitable for biophysical studies with high ATPase activity. The success of this mutagenesis strategy can be seen by comparing the final CL-Pgp, which conveyed high levels of yeast fungicide resistance, with the previously constructed all-Ala-CL mutant. The latter produced minimal drug resistance when expressed in yeast (Figure 6B). These results show that directed evolution successfully produced a novel CL Pgp that bypassed the limitations of the previously constructed mouse and human CL Pgps [16,17,22,30], as it is both active in yeast functional assays and can be readily purified in milligram quantities.

The site-saturation mutagenesis strategy used in this study replaced each of the six intracellular Cys with a fully degenerate codon that encoded all 20 standard amino acids, including Cys. Although Cys was allowed in this strategy, Cys revertants were only found abundantly at two positions in the linker region (Cys^638^ and Cys^669^), suggesting that other amino acids support Pgp function and could directly compete with Cys during selection (Figures 3 and 4). In general, Gly and Ser were the most prominent substitutions at multiple native Cys positions, contrary to the BLOSUM62 (BLOcks of Amino Acid SUbstitution Matrix) matrix where Gly carries one of the highest penalties (−3) for Cys replacement, while Ser is considered a more conservative substitution (penalty=−1) [45]. In a similar directed evolution study, Ullman and co-workers found that site saturation mutagenesis of Cys produced substitutions with small non-polar amino acids and also a prevalence of larger non-polar residues such as Leu and Met (penalty=−1) [29]. We would argue that the prevalence for a given substitution is likely dependent on the location of the Cys within a given protein. In Pgp for example, Gly and Ser most frequently replaced Cys residues in the glycine-rich Walker A motifs (Cys^427^ and Cys^1070^). These two Cys are highly conserved among Pgp orthologues (Supplementary Table S2 at http://www.bioscirep.org/bsr/034/bsr034e116add.htm), but both Gly and Ser are naturally prevalent at these two positions in other ABC transporters [46].

Among the unique mutants, Cys was often substituted by small non-polar or polar amino acids that likely maintain or enhance the flexibility at the Cys position. Cys^1121^ of NBD2 was an exception to this trend, as Arg, a large positively charged amino acid, was highly preferred at this site. The Pgp structure suggests that this Arg would be in close proximity to a negatively charged residue (Glu^269^) in the ICL2 that crosses over from the N-terminal TMD (blue) and docks into NBD2 (green; Figures 1 and 4B). This may explain the preference for Arg, which may form a salt bridge that would stabilize domain interactions. A salt bridge within ICL2 (between Arg^276^ and Glu^256^) was recently found to be essential for maturation of human Pgp (ref) indicating that proper folding is necessary for docking of this loop into NBD2 [47]. Maintaining or even strengthening domain interactions can be crucial for assembly of multidomain proteins and their maturation to the cell surface as has been demonstrated for the related ABC transporter CFTR (cystic fibrosis transmembrane conductance regulator). For example, V510D in NBD1 of CFTR can form a salt bridge with Arg^1070^ in ICL4 (crossing-over from the C-terminal TMD), promoting maturation and trafficking to the cell surface, and thereby can rescue the folding defect of the disease causing mutation ΔF508 [48,49].

Surprisingly, the two native Cys positions (Cys^638^ and Cys^669^) that showed many revertants are both located in the linker that connects the N-terminal NBD to the C-terminal TMD, which is considered to be highly flexible with little secondary structure (Figure 1) [6]. The amino-acid sequence of the linker, including these two Cys, is not conserved among ABC transporters or Pgp orthologues, for example Asp and His are found in human Pgp (Supplementary Table S2 at http://www.bioscirep.org/bsr/034/bsr034e116add.htm). Previous work has demonstrated that human Pgp maintains some level of drug stimulated ATPase activity when the two halves of Pgp are coexpressed without being connected by the linker, but proper protein expression is severely impaired [50]. Additional work suggests that the linker may also have a regulatory function in Pgp [51]. The tendency to retain Cys at these two positions may indicate some importance for these two polar residues in mouse Pgp, while other orthologues prefer charged Asp (34%) and Arg (49%) residues. The fact that Cys reversions occurred at these positions to a major extent indicates that site-saturation mutagenesis and stringent mutant selection was able to detect and successfully replace the Cys positions that were more difficult to substitute.

Although mutants generated by directed evolution were active, we did observe some variation in the degree of drug resistance among the C6Z mutants (Figure 5). The differing performance of the mutants between doxorubicin, FK506, and valinomycin may be explained by the mechanisms of action of these compounds. FK506, a calcineurin signalling inhibitor, and valinomycin, an ionophore, likely have more transitory effects on the physiology of the yeast cells than doxorubicin, which intercalates into DNA and interferes with DNA replication even at low intracellular concentrations [52]. Therefore doxorubicin may be more sensitive to assess how efficient Pgp mutants export drugs and prevent their intracellular accumulation.

In summary, this study demonstrated that directed evolution successfully yielded a CL Pgp that is well expressed, completely active in *in vivo* yeast assays, and can be readily purified. Maintaining activity in yeast functional assays is a critical improvement of our new CL-Pgp that allow us to rapidly screen activity of new mutants where Cys are reinserted at strategic positions for biophysical studies. Furthermore, analysis of the frequency of amino-acid substitutions at a given Cys position, suggests that substitutions are highly dependent on the local environment of the native Cys. Our screen identified novel non-conservative Cys substitutions and revealed important, previously unnoticed domain interactions around Cys^1121^. The information gained from this study may be used to guide the construction of other CL proteins where Ala and Ser have proven unsuccessful.

## Online data

Supplementary data
