# Supplementary material for: Directed evolution of P-glycoprotein cysteines reveals site-specific, non-conservative substitutions that preserve multidrug resistance
Source: Biosci Rep. 2014 Jun 25;34(3):e00116. doi: 10.1042/BSR20140062 (PMC4069687; doi:10.1042/BSR20140062)
Supplement: Supplementary data [file bsr034e116add.pdf]

Biosci. Rep. (2014) / **34** / art:e00116 / doi 10.1042/BSR20140062

OPEN ACCESS

Original Paper

Bioscience Reports

www.bioscirep.org

Bioscience Reports

SUPPLEMENTARY DATA

Directed evolution of P-glycoprotein cysteines reveals site-specific, non-conservative substitutions that preserve multidrug resistance

Douglas J. SWARTZ*†[1], Leo MOK*†[1], Sri K. BOTTA*†, Anukriti SINGH*†, Guillermo A. ALTENBERG†‡ and Ina L. URBATSCH*†[2]

*Department of Cell Biology and Biochemistry, Texas Tech University Health Sciences Center, Lubbock, Texas, U.S.A.
†Center for Membrane Protein Research, Texas Tech University Health Sciences Center, Lubbock, Texas, U.S.A.
‡Department of Cell Physiology and Molecular Biophysics, Texas Tech University Health Sciences Center, Lubbock, Texas, U.S.A.

Table S1 Oligonucleotide sequences used for site-saturation mutagenesis and site-directed mutagenesis of single TM Cys mutants

Only the forward mutagenic primer sequences are shown. Reverse primer sequences are identical but complimentary to the forward primer.

Primer name	Sequence (5′ to 3′)
Mutagenic primers	
C427-NNN	CGGAAATTCTGGTNNNGGCAAGTCCAC
C638-NNN	GGAAATGAGGCTNNNAAGTCTAAGGACGAG
C669-NNN	CACTAGAAAGTCCATTNNNGGTCCACACGATC
C1070-NNN	GGTTCTTCCGGTNNNGGCAAGTCCACTG
C1121-NNN	CTATTCTTTTCGACNNNAGCATTGCAGAG
C1223-NNN	CCAGAGAAGGAAGAACTNNNATCGTCATTGCTC
C133A	TCCTTCTGGGCCCTGGCCGCTGGAAGACAAATCCACAAGATTAGACAGAAG
C713A	GGTATCTTCGCTGCCATCATTAACGGTGGTTTGCAGCCAGCTTTTTCTGTG
C952A	TACGCTGCCGCATTCAGATTCGGTGCTTATTTGGTGACTCAGCAATTGATG
Flanking primers	
NBD1/linker	CCTTGTTTCTTTTTCTGCACAA (Forward)
	CTGTCTTTGGGTTTCTGGTGGACCACC (Reverse)
NBD2	CCATACCGTAACGCTATGAAAAAGGCTC (Forward)
	GAAGTGTCAACAACGTATCTACCAACG (Reverse)

[1] These authors contributed equally to this work.
[2] To whom correspondence should be addressed (email ina.urbatsch@ttuhsc.edu).

© 2014 The Author(s) This is an Open Access article distributed under the terms of the Creative Commons Attribution Licence (CC-BY) (http://creativecommons.org/licenses/by/3.0/) which permits unrestricted use, distribution and reproduction in any medium, provided the original work is properly cited.

Table S2 Relative frequency of each amino acid found at the nine native Cys positions in closely related Pgp orthologs

Position	Cys133*	Cys427	Cys638	Cys669	Cys713	Cys952	Cys1070	Cys1121	Cys1223
A									
C	84	100	11	5	98	98	100	96	98
D			34						
E			5						
F								4	
G	2		23						
H				23					
I									
K				16					
L	2								
M									
N			7						
P									
Q				2					
R				49					2
S			14	2	2	2			
T	11			2					
V									
W									
Y			7						
Total%	100	100	100	100	100	100	100	100	100

*Human and mouse Pgp were aligned with 46 closest related orthologs (⩾70% sequence identity) from the Ensembl database. The relative frequency (%) of amino acids found at each Cys position is shown.

Received 15 April 2014/5 May 2014; accepted 6 May 2014

Published as Immediate Publication 14 May 2014, doi 10.1042/BSR20140062

© 2014 The Author(s) This is an Open Access article distributed under the terms of the Creative Commons Attribution Licence (CC-BY) (http://creativecommons.org/licenses/by/3.0/) which permits unrestricted use, distribution and reproduction in any medium, provided the original work is properly cited.
